# Influence of micro- and nanoscale cues on immune factors secretion: implications for immunomodulation

**DOI:** 10.1093/rb/rbaf130

**Published:** 2025-12-18

**Authors:** Zhiling Luo, Yulian Zheng, Wentao Lin, Liang Zhang, Xinyi Huang, Yongli Li, Yushan He, Xiao Shen, Hengshu Zhang, Wei Huang, Wenguo Cui, Lu Chen

**Affiliations:** Department of Burn and Plastic Surgery, The First Affiliated Hospital of Chongqing Medical University, Chongqing 400016, P.R. China; Department of Emergency, The First Affiliated Hospital of Chongqing Medical University, Chongqing 400016, P.R. China; Department of Burn and Plastic Surgery, The First Affiliated Hospital of Chongqing Medical University, Chongqing 400016, P.R. China; Department of Burn and Plastic Surgery, The First Affiliated Hospital of Chongqing Medical University, Chongqing 400016, P.R. China; Department of Ultrasound, The First Affiliated Hospital of Chongqing Medical University, Chongqing 400016, P.R. China; Department of Burn and Plastic Surgery, The First Affiliated Hospital of Chongqing Medical University, Chongqing 400016, P.R. China; Department of Burn and Plastic Surgery, The First Affiliated Hospital of Chongqing Medical University, Chongqing 400016, P.R. China; Department of Burn and Plastic Surgery, The First Affiliated Hospital of Chongqing Medical University, Chongqing 400016, P.R. China; Department of Burn and Plastic Surgery, The First Affiliated Hospital of Chongqing Medical University, Chongqing 400016, P.R. China; Department of Burn and Plastic Surgery, The First Affiliated Hospital of Chongqing Medical University, Chongqing 400016, P.R. China; Chongqing Municipal Health Commission, Key Laboratory of Musculoskeletal Regeneration and Translational Medicine, Chongqing 400016, China; Department of Orthopaedics, Shanghai Key Laboratory for Prevention and Treatment of Bone and Joint Diseases, Shanghai Institute of Traumatology and Orthopaedics, Ruijin Hospital, Shanghai Jiao Tong University School of Medicine, Shanghai 200025, China; Department of Burn and Plastic Surgery, The First Affiliated Hospital of Chongqing Medical University, Chongqing 400016, P.R. China

**Keywords:** micro- and nanofibers, scale-dependent effect, immune microenvironment, immune factor secretion, tissue engineering materials

## Abstract

Immune factors secreted by immune cells play a pivotal role in orchestrating inflammatory responses and facilitating tissue regeneration. Fiber dressings, owing to their extracellular matrix-like architecture and tunable physical properties, have emerged as promising candidates in regenerative medicine. Beyond serving as passive structural supports, fibers are increasingly recognized as active modulators of cell behavior through their inherent physical characteristics. However, how fiber diameter at the micro- and nanoscale influences the immune factor secretion profile of immune cells remains poorly defined. In this study, three types of fibers with distinct diameter scales were fabricated to systematically assess their immunomodulatory effects. *In vitro* analyses revealed that microscale fibers markedly enhanced the secretion of pro-regenerative and anti-inflammatory factors, such as VEGF, EGF, IL-10 and TGF-β1, while suppressing pro-inflammatory factors including TNF-α and IL-6. Mechanistic investigations indicated that this size-dependent immunomodulation may be driven by activation of the FAK-Wnt signaling pathway triggered by topographical cues. *In vivo*, microscale fibers significantly promoted neovascularization, attenuated inflammatory responses and accelerated tissue repair, further corroborating their immunoregulatory potential in a physiological setting. These findings establish fiber diameter as a critical physical cue for shaping the immune microenvironment and present a new strategy for immunoregulation through structural design. This work provides a conceptual framework for the development of biomaterials with intrinsic immunomodulatory properties and offers new therapeutic insights for the treatment of chronic inflammation-associated disorders.

## Graphical Abstract

**Figure:**
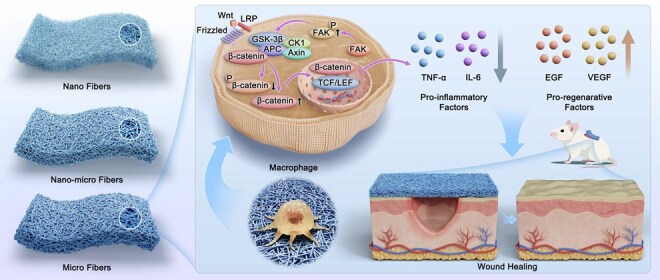

## Introduction

The pivotal role of interactions between biomaterials and the immune system in guiding tissue repair and regeneration has gained increasing recognition [1, 2]. As a critical component of tissue engineering, biomaterials are no longer regarded merely as passive structural supports, but as active regulators capable of dynamically modulating the regenerative microenvironment through their interplay with immune system [3]. This mechanism is critical across diverse applications, including tissue repair, tumor immunotherapy and artificial organ integration [4]. Current research largely focuses on biomaterials as carriers for bioactive factors or drugs to influence immune responses and reshape the local microenvironment [5–7]. However, compared to exogenous interventions, the extent to which the intrinsic physical properties of biomaterials—such as surface topography, mechanical properties and scale—can directly regulate the immune microenvironment remains underexplored. Therefore, understanding the intrinsic relationship between material physical characteristics and the immune microenvironment is essential for the rational design of immunomodulatory biomaterials.

Fiber dressing, as a prototypical class of biomimetic materials, has garnered widespread attention in tissue regeneration due to its structural resemblance to the native extracellular matrix (ECM) [8, 9]. Their mesh-like architecture provides a high surface-area-to-volume ratio, excellent biocompatibility and tunable physical properties [10]. Among these, fiber diameter scale is a key physical attribute that has demonstrated significant regulatory effects on various cell types involved in tissue repair. For example, Wang *et al*. [11] showed that intermediate-diameter fibers promote aligned neurite outgrowth, while larger fibers further facilitate Schwann cell migration. Likewise, Milleret *et al.* [12] found that, compared to ultrafine fibers, finer fibers enhance endothelial cell adhesion and proliferation, suggesting a role for fiber dimension in angiogenesis. Despite growing evidence of fiber diameter scales influencing cellular behavior, its immunomodulatory effects remain poorly characterized. Given the central role of the immune microenvironment in orchestrating inflammation resolution and tissue regeneration, a comprehensive understanding of how fiber diameter scale affects immune responses is of both theoretical significance and translational relevance [13–16].

The immune microenvironment is fundamentally defined by the spatiotemporal expression patterns of various immune factors [17–19]. These factors orchestrate the functional states of immune cells, guiding the inflammatory process from initiation to resolution and ultimately promoting tissue reconstruction [20]. For instance, sustained overexpression of pro-inflammatory factors such as TNF-α and IL-6 can lead to chronic inflammation and tissue damage [21, 22]. In contrast, anti-inflammatory factors like IL-10 and TGF-β1 help suppress excessive immune responses and facilitate the resolution of inflammation [23]. Additionally, pro-regenerative factors such as EGF and VEGF contribute to cell migration, angiogenesis and tissue remodeling [24, 25]. Macrophages, as major producers of these immune mediators, play a pivotal role in damage sensing, inflammatory regulation and repair progression [26–28]. Their secretory profiles directly shape the characteristics of the immune microenvironment and influence the quality of tissue regeneration [29, 30]. Therefore, elucidating how fiber diameter affects the immune factor secretion patterns of macrophages could uncover the immunomodulatory potential of fibers and provide a theoretical foundation for the design of immuno-regulatory regenerative materials.

In chronic diabetic wounds, macrophage dysfunction often leads to aberrant expression profiles of immune mediators, characterized by sustained overexpression of pro-inflammatory cytokines and insufficient levels of anti-inflammatory and pro-regenerative factors [31]. This imbalance creates a persistent pro-inflammatory microenvironment that significantly impairs tissue regeneration. In this study, we fabricated three types of fibers with distinct diameter scales to systematically investigate their regulatory effects on immune factor secretion by macrophages. We focused on the differential expression of pro-inflammatory factors such as TNF-α and IL-6, as well as pro-regenerative and anti-inflammatory factors including VEGF, EGF, IL-10 and TGF-β1, aiming to elucidate how the physical scale of biomaterials modulates macrophage-mediated immune factor secretion and thereby influences the transition of the immune microenvironment. By exploring the interaction between fiber micro- and nanoscales and immune factor secretion in the absence of exogenous bioactive agents, this study highlights the intrinsic capacity of scaffold architecture to modulate immune responses. These findings provide both theoretical rationale and experimental evidence for the development of regenerative biomaterials with immunomodulatory functions.

## Materials and methods

### Preparation of micro- and nanofibers

Fibers with different micro- and nanoscale cues were prepared using an electrospinning device. Poly (L-lactic acid) (PLLA; 100 kDa; Daigang, China) was dissolved in hexafluoroisopropanol (≥99% purity, Sigma, USA) to obtain homogeneous solutions by continuous magnetic stirring for 12 h at room temperature, with concentrations of 8%, 12% and 16% (w/v), respectively. The electrospinning process was performed under controlled environmental conditions (25°C, relative humidity 45–55%), with an applied voltage of 12 kV and a flow rate of 0.03 mL/min. The resulting micro- and nanofibers (MNFs) were collected on an aluminum foil-covered rotating drum positioned 15 cm from the needle tip and vacuum-dried overnight to remove residual solvent.

### Characterization of MNFs

Scanning electron microscopy (SEM, Hitachi, Japan) was utilized to characterize the morphology and surface scales of the fibers. Before imaging, a thin gold coating was applied to the samples via sputtering. The average diameters of the MNFs were calculated from SEM images using ImageJ software, with at least 50 fibers randomly measured for each group to ensure statistical reliability.

To evaluate the degradation behavior of the MNFs, the fibers were cut into specimens of uniform size (diameter 15 mm, thickness 1 mm) and immersed in 1× PBS (Gibco, USA) at 37°C under gentle shaking. At predetermined time points (1, 2 and 3 weeks), samples were collected, rinsed with deionized water and vacuum-dried. The morphology of the degraded samples was observed under SEM, and the mass loss percentage was calculated as follows: (initial mass−residual mass)/initial mass×100%.

The mechanical properties of the MNFs were assessed using an electronic universal testing machine (MTS, USA). Rectangular specimens (10 mm×30 mm, *n* = 5 per group) were cut from the fibers and tested under uniaxial tensile mode at a crosshead speed of 5 mm/min until failure. Stress–strain curves were recorded.

### Biocompatibility of MNFs

RAW 264.7 murine macrophages (ATCC, USA) were used in this study. Cells were cultured in specialized medium (Procell, China) with 10% (v/v) fetal bovine serum and 1% (v/v) penicillin–streptomycin at 37°C under 5% CO_2_. Passaging was conducted every 2–3 days upon reaching 70–80% confluence.

Macrophages were seeded onto MNFs with three different fiber diameters. After 3 days of culture, the cells were fixed, dehydrated through graded ethanol and dried using the critical point method. A thin gold layer was then applied before Bioelectron microscopy (Bio-EM) observation of cell adhesion and morphology. For cytotoxicity assessment, the culture medium was replaced with a Live/Dead staining solution (Sigma, USA), and cells were incubated at 37°C. Live and dead cells were then visualized under a fluorescence microscope, and the cell death rate was quantified. Macrophages seeded on MNFs were cultured for 24, 48 and 72 h, fixed with paraformaldehyde (PFA) and stained with DAPI. Cell adhesion and proliferation were observed using a fluorescence microscope. In addition, after 3 days of culture, macrophages were fixed with 4% (w/v) PFA and stained with Phalloidin (Sigma, USA) to visualize the actin cytoskeleton, while nuclei were counterstained with DAPI. The CCK-8 assay was performed in accordance with the manufacturer’s instructions to evaluate cell viability at 24, 48 and 72 h. The optical density at 450 nm was recorded.

### Immunofluorescence staining

To assess the inflammatory status of macrophages, immunofluorescence (IF) staining was performed to detect markers CD86 and CD206. Macrophages were seeded onto MNFs in 24-well plates and incubated at 37°C with 5% CO_2_ for 3 days. Fixation, permeabilization and blocking steps were carried out sequentially using PFA for 15 min, 0.5% (v/v) Triton X-100 for 10 min and goat serum for 1 h at room temperature, respectively, to ensure optimal sample preparation. The primary antibodies for CD86 (Proteintech, China) and CD206 (CST, USA) were incubated at 1:200 dilution overnight at 4°C. After three washes with PBS, a 1-h incubation at room temperature with appropriate fluorescently labeled secondary antibodies was performed, followed by nuclear counterstaining with DAPI for 10 min. Fluorescence images were captured using a confocal laser scanning microscope (CLSM) with identical imaging settings for all groups to ensure comparability.

### Immune factor secretion analysis

Immune factor production by macrophages cultured on fibers with distinct diameter scales features was quantitatively assessed using ELISA. Macrophages were seeded onto nanofibers in 24-well plates and incubated at 37°C with 5% CO_2_. Following a 3-day culture period, macrophages were transferred to serum-free, high-glucose DMEM (Gibco, USA) and incubated for an additional 24 h to facilitate cytokine secretion. The supernatants were collected and centrifuged for 10 min to remove cell debris. The concentrations of key immune factors, including IL-6, TNF-α, VEGF, EGF, IL-10 and TGF-β1 were determined according to the protocols provided by the ELISA kit manufacturers.

### Western blotting

Western blotting (WB) analysis was conducted to examine the expression of CD86 (Proteintech, China), CD206 (CST, USA), ARG1 (CST, USA), FAK (CST, China), p-FAK (CST, China), β-catenin (CST, USA), p-β-catenin (CST, USA) and β-actin (CST, USA). After 3 days of culture on nanofibers under standard conditions (37°C, 5% CO_2_), macrophages in 6-well plates were subjected to lysis in RIPA buffer (Beyotime, China) fortified with both protease and phosphatase inhibitors. The lysates were centrifuged (4°C, 15 min), and the resulting supernatants were analyzed for protein concentration by a subsequent BCA assay. Equal protein quantities (20 μg) were separated by SDS–PAGE and transferred onto polyvinylidene fluoride membranes. Following a 30-min incubation in quick blocking solution, membranes were exposed to primary antibodies at 4°C overnight. After TBST, secondary antibody treatment was conducted for 1 h. Protein signals were visualized by enhanced chemiluminescence (Beyotime, China) and analyzed using ImageJ.

### Reverse transcription quantitative polymerase chain reaction

RNA was extracted from the cells with TRIzol reagent (Epizyme, China) following the manufacturer’s protocol. RNA quality and quantity were assessed by NanoDrop spectrophotometry (Thermo, USA), with A260/A280 ratios of 1.8–2.0. From total RNA, complementary DNA was synthesized using a SuperMix (MCE, USA) following the provided protocol. Reverse transcription quantitative polymerase chain reaction (RT-qPCR) was performed to analyze the expression levels of IL-6, TNF-α, CD206 and ARG1 in macrophages cultured on MNFs of different diameter scales, with GAPDH used as the internal reference gene for normalization. Reactions were run in triplicate on a Real-Time PCR System (Biorad, USA) using SYBR Green Master Mix (MCE, USA). Gene expression levels were quantified by applying the 2^−ΔΔCt^ method, with all primer sequences listed in Table 1.

**Table 1:** Primer sequence.

Primer	Forward (5′ to 3′)	Reverse (5′ to 3′)
GAPDH	TGTGTCCGTCGTGGATCTGA	TTGCTGTTGAAGTCGCAGGAG
TNFα	CGCTGAGGTCAATCTGC	GGCTGGGTAGAGAATGGA
IL-6	ACAGAAGGAGTGGCTAAGGA	AGGCATAACGCACTAGGTTT
ARG1	ACGGTCTGTGGGGAAAG	TCAGGGGAGTGTTGATGTC
CD206	CAAGCGATGTGCCTACC	AATGCTGTGGATACTTGCC

### Animal experiments

The Animal Experimental Ethics Committee of Chongqing Medical University approved all experimental procedures (Approval No. 2024-0384). A diabetic rat model was used to evaluate wound healing. Male SD rats, aged 8–10 weeks and weighing 200–250 g, were maintained under standard laboratory conditions with *ad libitum* access to food and water. Rats were rendered diabetic by a single intraperitoneal administration of streptozotocin (STZ, 60 mg/kg) freshly prepared in 0.1 M citrate buffer (pH 4.5). Blood glucose levels were measured from the tail vein on Days 3, 7 and 14 post-injection, and rats with blood glucose consistently above 16.7 mmol/L were considered diabetic and included in the study. Full-thickness dorsal skin wounds (10 mm in diameter) were generated in rats anesthetized with 1% pentobarbital sodium (50 mg/kg) via intraperitoneal injection. The wounds were randomly assigned into four groups: untreated control and three kinds of fibers. Sterilized circular nanofiber patches (diameter 10 mm, thickness 0.1 mm) were applied to the wounds in the respective groups, while the control group received no treatment. All animals were monitored daily for general health, behavior and signs of infection throughout the experimental period.

The wound healing progression was assessed both qualitatively and quantitatively. Digital photographs of the wounds were taken at predetermined time points (Days 0, 3, 7, 14 and 21) using a standardized camera setup under consistent lighting conditions. A scale ruler was included in each image to allow accurate size calibration. For quantitative analysis, the wound boundaries were manually delineated in ImageJ. The wound margins were manually traced to calculate the area at each time point. Relative area of wounds was determined by normalizing each wound area to its baseline size.

Tissue samples were collected from the wound edges at Days 3, 7 and 21, fixed in PFA for 24 h, dehydrated through an ethanol gradient and embedded in paraffin. Serial sections were prepared using a microtome. Sections from Days 3 and 7 were prepared for IF staining by deparaffinization, graded ethanol rehydration and antigen retrieval in citrate buffer at 95°C for 20 min. After BSA block, sections were incubated overnight with primary antibodies against CD86 (Proteintech, USA) and CD206 (CST, USA), followed by 1-h incubation with fluorescence-conjugated secondary antibodies at room temperature. Nuclei were visualized by DAPI staining. Hematoxylin and eosin (H&E) staining was conducted on Day 3 and Day 21 sections following standard protocols, including hematoxylin staining and eosin, and sections were dehydrated and mounted with neutral resin. Masson’s trichrome staining was performed on Day 21 sections according to the manufacturer’s instructions (Solarbio, China) to visualize collagen deposition. IF staining for CD31 (AiFang biological, China) was performed on Day 21 sections using the same procedures as above to evaluate angiogenesis. All samples were analyzed using microscopy and ImageJ software.

### Statistical analysis

All data are presented as mean±SD. Statistical analysis was carried out using one-way ANOVA, with significance defined at *P* < 0.05. All analyses were performed using GraphPad Prism and Origin. All relevant *P*-values are shown in the corresponding bar graphs.

## Results

### Characterizations of MNFs

**Figure 1:**
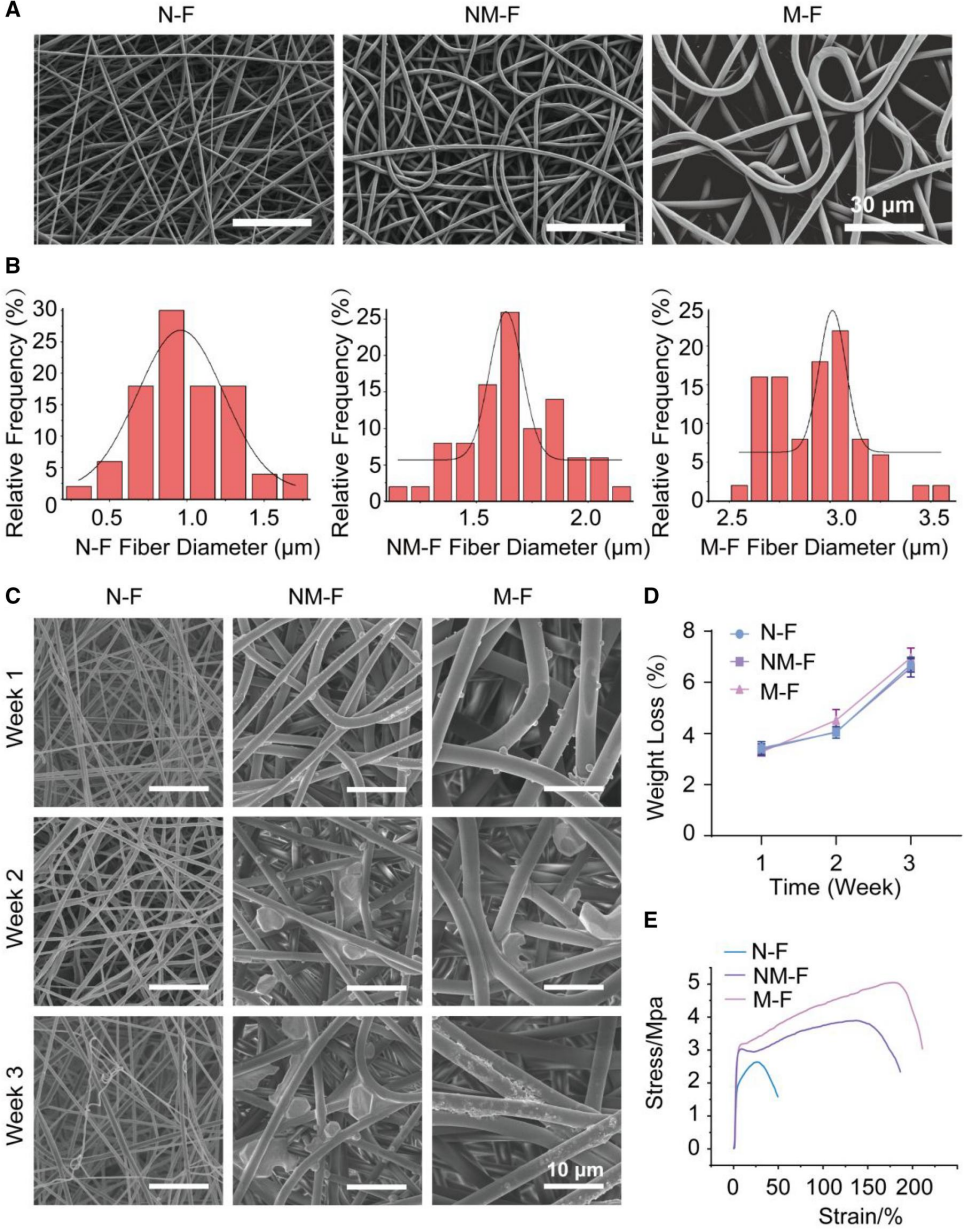
Characterization of MNFs, including morphology, degradation behavior and mechanical properties. (**A**) SEM images illustrating the morphology of the MNFs with different fiber diameters. (**B**) Quantitative analysis of fiber diameters measured by ImageJ software. (**C**) Representative SEM images showing morphological changes of MNFs after degradation at different time points. (**D**) Weight loss of MNFs during the degradation process at Weeks 1, 2 and 3. (**E**) Stress–strain curves obtained from tensile testing, demonstrating the mechanical properties of the MNFs.

The fibers with different diameter scales were synthesized using electrospinning equipment with different PLLA solutions as raw materials. As shown in Figure 1A, SEM images revealed that increasing the PLLA concentration led to a progressive increase in fiber diameter. Fibers obtained from 8% and 16% PLLA solutions were designated as nano fibers (N-F) and micro fibers (M-F), respectively, while fibers derived from the 12% solution, exhibiting intermediate diameters, were termed nano-micro fibers (NM-F). Fiber diameter was quantitatively analyzed using ImageJ software, and the average diameters of N-F, NM-F and M-F were determined to be 955.02 ± 50.36 nm, 1632.50 ± 18.82 nm and 3010.72 ± 30.78 nm, respectively (Figure 1B). Collectively, these results confirmed the successful fabrication of fibers spanning three distinct diameter scales.

The degradation experiments revealed that the fibers in all three groups of MNFs exhibited some degree of bending and deformation, along with slight particle precipitation. The fibers showed weight loss over time. For N-F, the weight loss was 3.43 ± 0.25%, 4.04 ± 0.22% and 6.69 ± 0.30% at Weeks 1, 2 and 3, respectively. For NM-F, the corresponding values were 3.37 ± 0.23%, 4.06 ± 0.10% and 6.57 ± 0.35%. For M-F, the values reached 3.28 ± 0.13%, 4.52 ± 0.41% and 6.96 ± 0.38% (Figure 1C and D). These results indicate that all MNFs showed controlled degradation and good stability, maintaining sufficient structural integrity to support tissue repair. As a wound dressing, the mechanical strength of MNFs is particularly important, as superior tensile properties help ensure that the fibers remain intact during body movements such as bending or stretching. As a result, N-F exhibited a tensile strength of 2.64 ± 0.22 MPa; NM-F showed 3.9 ± 0.31 MPa; while M-F achieved 5.05 ± 0.37 MPa. Experimental results showed that as the fiber diameter increased from N-F to M-F, tensile strength improved significantly (Figure 1E).

### Biocompatibility of MNFs to macrophages

**Figure 2:**
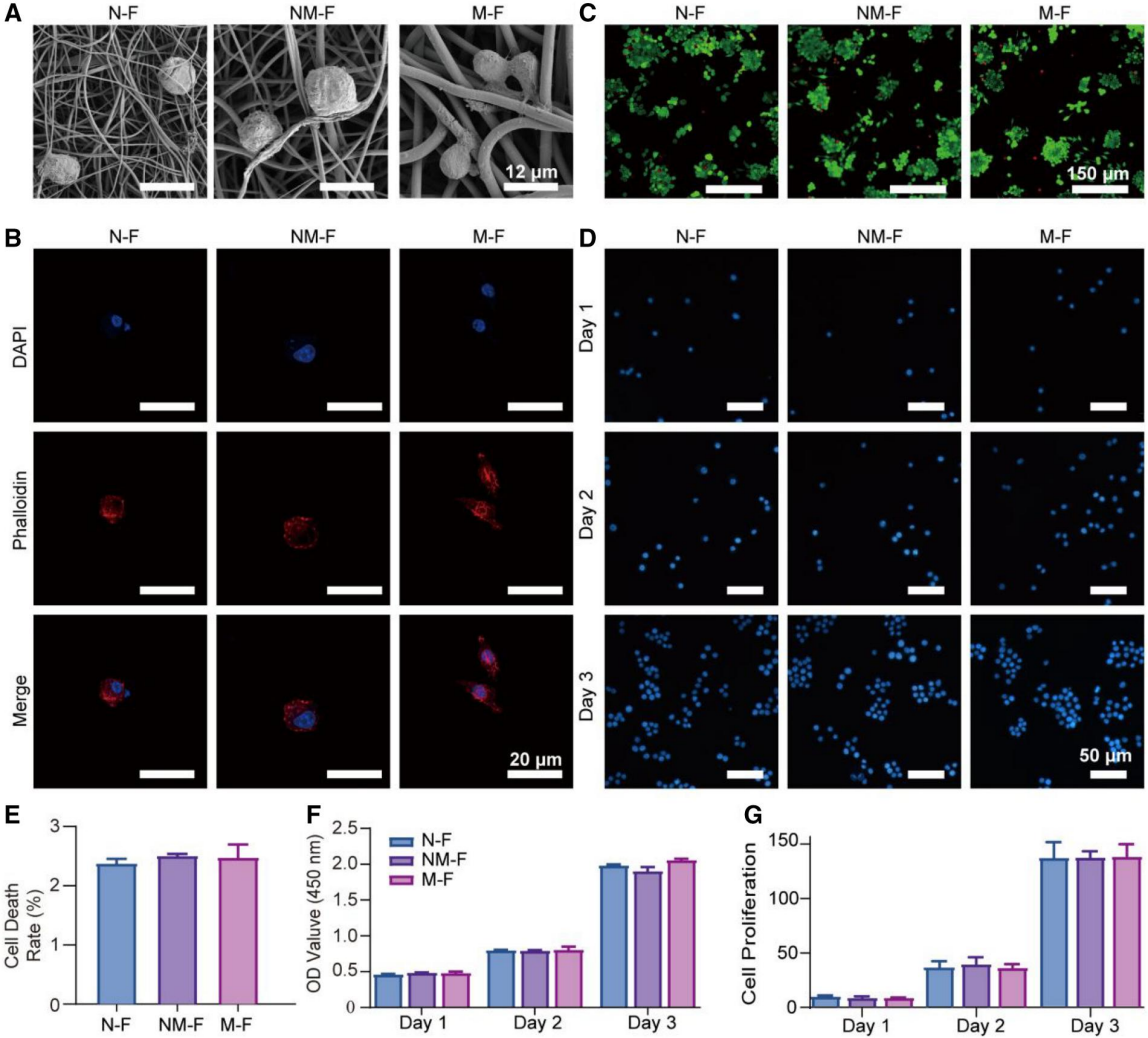
Biocompatibility of MNFs. (**A**) Bio-EM micrographs showing the morphology and adhesion of macrophages cultured on N-F, NM-F and M-F surfaces at Day 3. (**B**) CLSM images of macrophages stained with DAPI and phalloidin after 3 days culture on MNFs. (**C**) Live/dead fluorescence staining images of macrophages cultured on MNFs for 3 days. (**D, G**) Nuclear fluorescence staining of macrophages cultured on MNFs at Days 1, 2 and 3, observed by fluorescence microscopy and quantity statistics. (**E**) Quantitative analysis of cell death rate based on live/dead staining. (**F**) CCK-8 assay results showing the OD values at 450 nm, which reflect the cell viability of macrophages cultured on MNFs at Days 1, 2 and 3.

Through Bio-EM and cytoskeletal staining experiments, it was demonstrated that macrophages were able to successfully adhere to the three sets of MNFs (Figure 2A and B). According to Figure 2C and E, evaluating the biocompatibility of the three types of MNFs, all of them had low cell death rates, with the advantages of good biocompatibility and low toxicity. Cell metabolic activity was evaluated using the CCK-8 assay on Days 1, 2 and 3. The results showed good cell viability. In a separate set of samples, nuclear staining was performed on the same days to assess cell adhesion and proliferation. Macrophages were able to adhere to the MNFs of three different scales and exhibited time-dependent growth, with no significant differences observed among the groups (Figure 2D, F and G).

### Immune factor secretion regulated by MNFs

**Figure 3:**
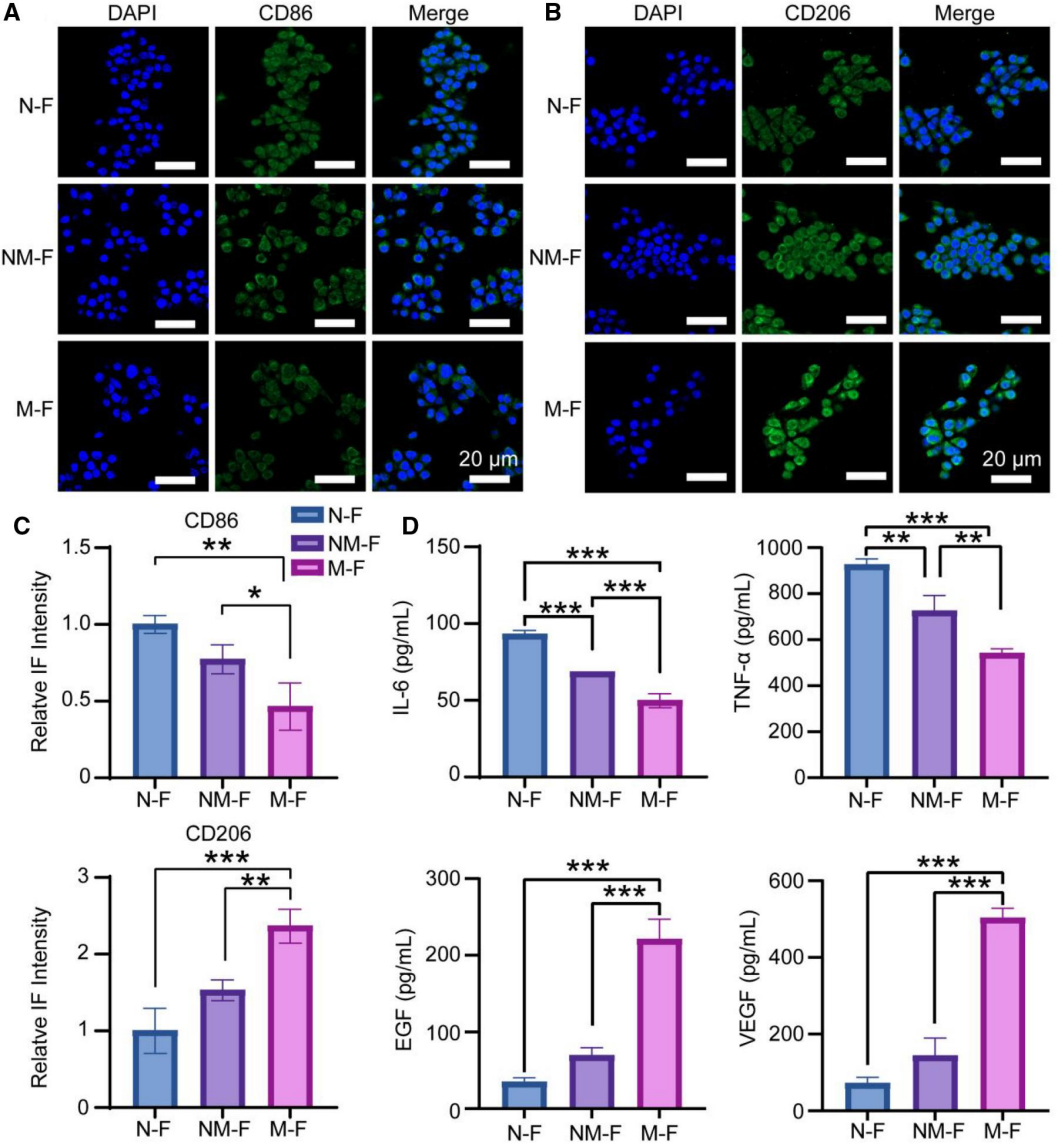
Impact of micro- and nanoscale regulates immune response and immune factor secretion. (**A–C**) IF analysis illustrating the expression levels of pro-inflammatory marker CD86 followed by anti-inflammatory marker CD206 in macrophage. (**D**) ELISA assessment of immune factor secretion, including TNF-α, IL-6, EGF and VEGF. *P < 0.05; **P < 0.01; ***P < 0.001.

As a biomaterial dressing, MNFs can significantly influence wound healing by modulating macrophage behavior. We employed CD86 and CD206 as markers to identify pro-inflammatory and pro-regenerative macrophages, respectively, and performed fluorescence staining on macrophages cultured on the three types of fibers. As shown in Figures 3A–C, fibers with larger diameters promoted a phenotypic shift of macrophages toward a pro-regenerative profile, as evidenced by increased CD206 expression and a corresponding decrease in CD86 expression.

Macrophages cultured on large-diameter fibers exhibited reduced levels of pro-inflammatory factors TNF-α and IL-6, and increased secretion of key wound-healing associated immune factors, including VEGF, EGF, IL-10 and TGF-β1. ELISA results confirmed that larger fiber diameters enhanced the production of these beneficial factors, indicating a shift toward a pro-regenerative rather than pro-inflammatory microenvironment (Figure 3D and Supplementary Figure S1).

### Mechanisms underlying immune factor secretion modulation

**Figure 4:**
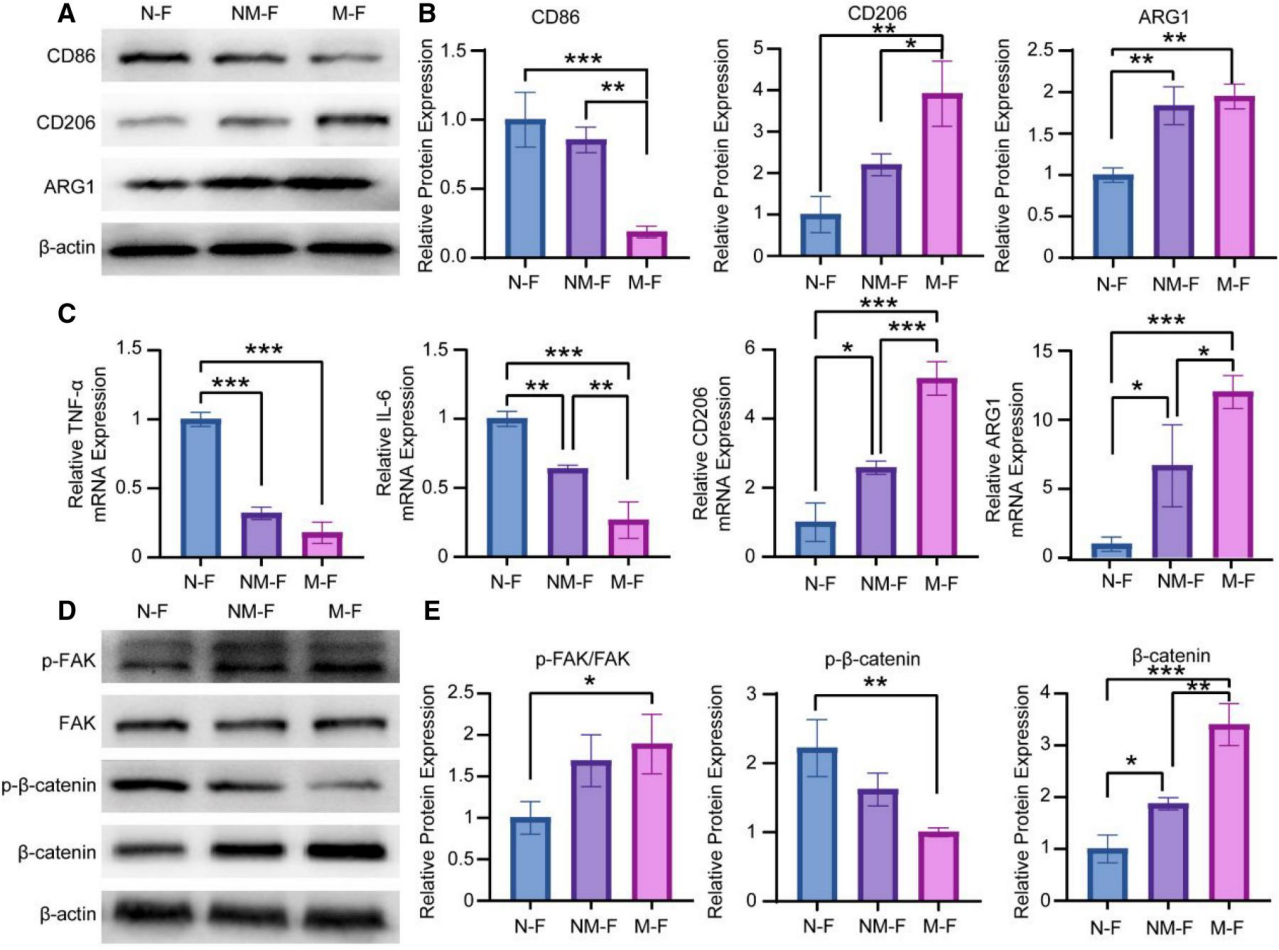
Macrophages immune factor secretion regulation-related signaling pathways. (**A**, **B**) Protein expression levels of CD86, CD206 and ARG1 in macrophages, evaluated via WB analysis. (**C**) RT-qPCR analysis of gene expression associated with macrophage polarization, including pro-inflammatory and anti-inflammatory markers, with results normalized to internal reference gene. (**D**, **E**) Analysis of protein expression within the FAK-Wnt signaling pathway in macrophages. *P < 0.05; **P < 0.01; ***P < 0.001.

WB analyses revealed that macrophages cultured on M-F exhibited increased expression of the anti-inflammatory markers CD206 and ARG1, while the pro-inflammatory marker CD86 was downregulated (Figure 4A and B). Meanwhile, RT-qPCR analysis showed that macrophages on N-F expressed higher levels of the pro-inflammatory genes TNF-α and IL-6, and lower levels of CD206 and ARG1, suggesting a more pro-inflammatory phenotype (Figure 4C). These results indicated that the diameter of MNFs influenced the phenotypic transition of macrophages, with larger-diameter fibers promoting a pro-regenerative phenotype that is more favorable for tissue repair.

To further understand the underlying signaling mechanisms, we investigated the FAK-Wnt pathway, which was significantly upregulated in macrophages cultured on larger-diameter fibers (Figure 4D and E). These results suggested that the FAK-Wnt pathway may mediate the polarization of macrophages toward a pro-regenerative phenotype in response to larger-diameter nanofibers.

### *In vivo* experimentation

**Figure 5:**
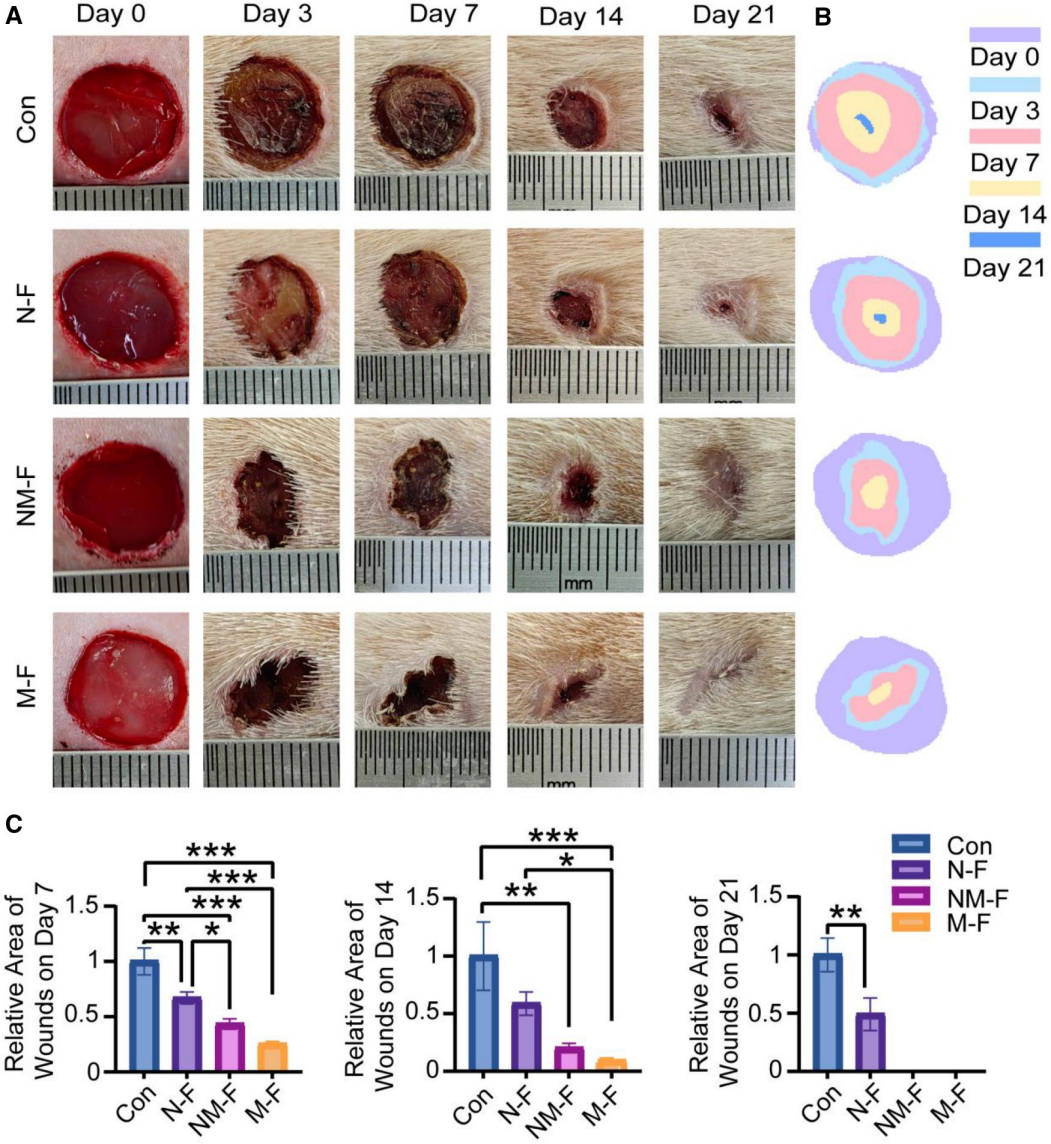
*In vivo* therapeutic efficacy of M-F in promoting wound healing. (**A**, **B**) Representative images of wounds showing that M-F treatment significantly enhanced wound closure over time *in vivo*. (**C**) Quantitative analysis of wound area, calculated using standardized image analysis software, with statistical comparison between control and treated groups at each time point. *P < 0.05; **P < 0.01; ***P < 0.001.

In diabetic rat models, wounds treated with MNFs exhibited significantly faster healing compared to the control group. At Day 21, wounds treated with M-F had fully healed, whereas the control group showed unhealing area (Figure 5A and B). Quantitative analysis of wound area revealed a marked reduction in size in fiber-treated groups, with the N-F group showing a small unhealing wound area and the NM-F and M-F groups achieving full closure on Day 21 (Figure 5C).

**Figure 6:**
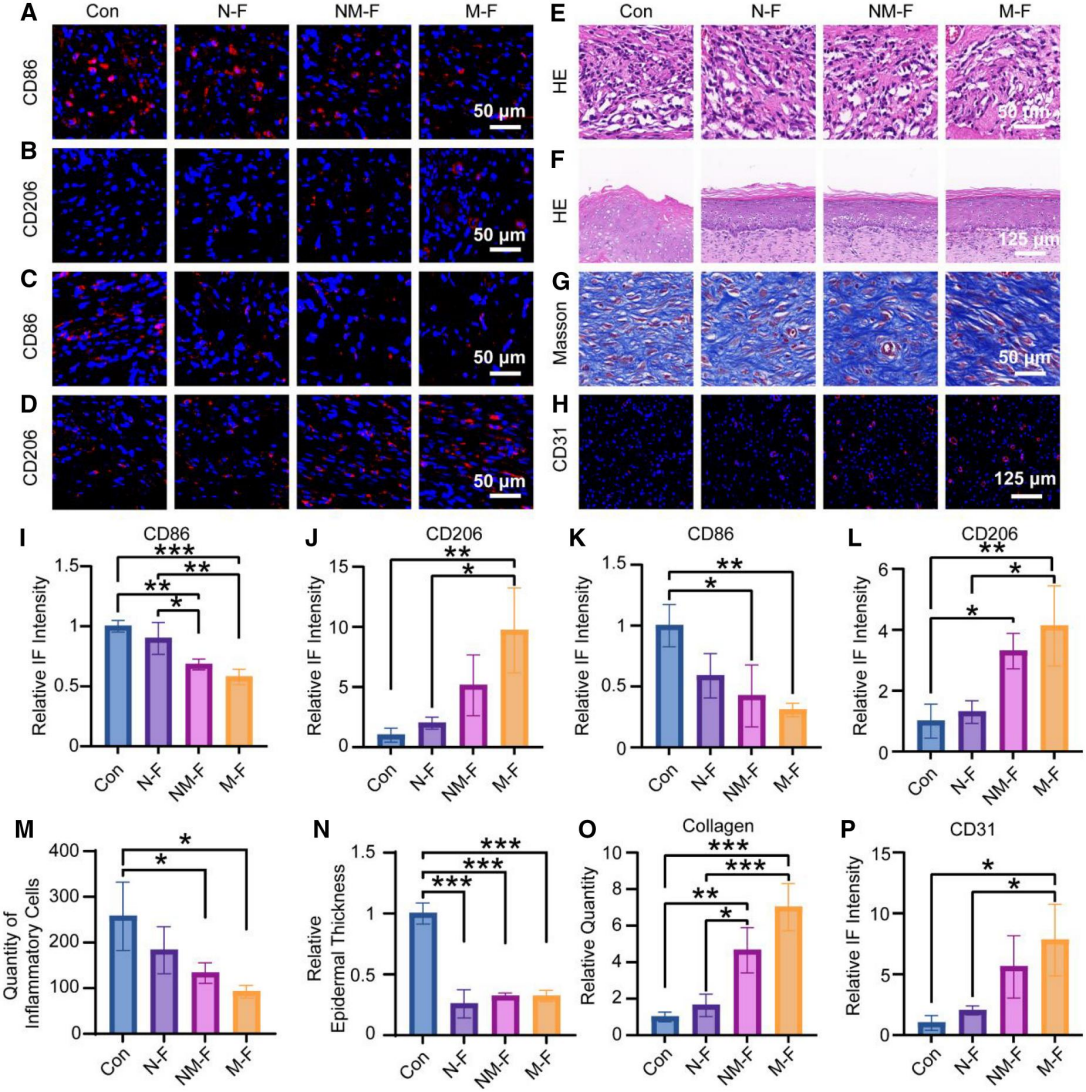
Histological analysis of *in vivo* wound healing. IF staining and quantitative analysis of macrophage markers CD86 and CD206 expression on (**A**, **B**, **I**, **J**) Day 3 and (**C**, **D**, **K**, **L**) Day 7, showing dynamic changes in macrophage polarization during wound healing. (**E**, **M**) H&E staining of wounds at Day 3 to evaluate initial tissue response and inflammatory cell infiltration. (**F**, **N**) H&E staining and analysis of epidermal thickness at Day 21. (**G**, **O**) Masson’s trichrome staining and statistical evaluation of dermal collagen deposition at Day 21. (**H**, **P**) IF staining and quantification of CD31 on Day 21 to assess neovascularization. *P < 0.05; **P < 0.01; ***P < 0.001.

Histological analysis further confirmed the superior healing efficacy of MNF-treated wounds. IF staining for CD86 and CD206 indicated that larger-diameter MNFs promoted macrophages polarizing toward the pro-regenerative phenotype (Figure 6A–D and I–L). H&E staining on Day 3 revealed reduced inflammatory cell infiltration in the M-F group compared to other groups (Figure 6E and M). H&E staining on Day 21 showed more uniform epithelial formation and Masson staining demonstrated denser collagen deposition in the M-F treated group compared to the control group (Figure 6F, G, N and O). Additionally, IF staining for CD31 revealed enhanced angiogenesis in the M-F group, with a 7.8-fold increase in new blood vessels compared to the control (Figure 6H and P).

These findings suggest that larger-diameter MNFs not only accelerate wound healing by enhancing macrophage polarization to the pro-regenerative phenotype but also promote critical processes such as collagen deposition and angiogenesis, ultimately leading to complete wound closure.

## Discussion

This study systematically evaluated how fiber diameter scale influences the immune factor secretion profiles of immune cells. The findings revealed that fiber diameter scale, as a key physical parameter, markedly regulates the expression of both pro-inflammatory and pro-regenerative factors. Unlike previous studies that primarily focused on cell phenotype or morphology [32], our analysis highlights a direct link between the microstructural properties of biomaterials and immune cell function, specifically in terms of immune factors secretion. These insights offer a new perspective for understanding the interaction mechanisms in the ‘biomaterial–immune’ system.

To elucidate the specific role of physical scale in immunomodulation, we fabricated three types of fibers with distinct average diameters: approximately 955.02 nm (N-F), 1632.50 nm (NM-F) and 3010.72 nm (M-F). All fibers exhibited favorable morphological stability and biocompatibility. Subsequent experimental analyses revealed that although macrophage proliferation showed no significant differences among the three scaffold types, their immune factor secretion profiles exhibited pronounced scale-dependent behavior. ELISA results demonstrated that the large-diameter M-F markedly downregulated pro-inflammatory cytokines TNF-α and IL-6, while upregulating repair-associated factors EGF and VEGF as well as anti-inflammatory cytokines IL-10 and TGF-β1. These findings suggest that M-F possess an inherent advantage in reshaping the immune microenvironment by promoting a shift from a pro-inflammatory to a pro-regenerative state. This evidence indicates that fiber diameter scale not only defines structural attributes but also actively participates in immune regulation as a form of mechanical cue. In contrast to conventional approaches relying on exogenous drugs or bioactive factors, the M-F effectively modulated macrophage secretion profiles through its intrinsic structural features. This scale-driven immunoregulatory mechanism offers enhanced biosafety and sustainability, highlighting the potential of structural design itself as a novel and precise strategy for immune intervention.

Materials with different diameter scales induce significant differences in immune factor secretion, but the mechanisms by which cells interpret and convert these physical cues into immune responses remain unclear. Therefore, we further investigated the molecular pathways underlying the regulation of macrophage function by material microstructure scales. Our study revealed that the scale-dependent immunomodulatory effects may rely on the coordinated activation of the FAK-Wnt signaling pathway. Specifically, the M-F likely enhances the density of cellular adhesion sites, thereby increasing mechanical stress transmission and activating the adhesion-associated protein FAK, which in turn induces cytoskeletal tension remodeling. Previous studies have demonstrated that cytoskeletal tension contributes to cell migration [33]. We further found that scale-induced alterations in cytoskeletal mechanics act as upstream triggers to sustain activation of the Wnt/β-catenin pathway, subsequently regulating the expression of multiple transcription factors closely associated with immune factor secretion. Ultimately, this cascade remodels macrophage functional states and their immune factor secretion profiles.

Given the close association between immune dysregulation and impaired tissue regeneration under chronic inflammatory conditions, we employed a diabetic rat wound model that closely mimics the immune imbalance characteristic of non-healing clinical wounds for *in vivo* validation. The *in vivo* results demonstrated that the M-F exhibited superior regenerative performance, significantly accelerating wound closure, suppressing inflammatory responses and promoting key regenerative processes such as angiogenesis and collagen deposition. In conjunction with *in vitro* findings, the enhanced tissue repair observed *in vivo* may be attributed to the consistent reprogramming of immune factor expression profiles induced by M-F. This immunomodulatory effect is likely a key driver of vascularization and matrix remodeling. The observed consistency between *in vitro* and *in vivo* outcomes reinforces the causal link between micro- and nanoscale structural cues, immune factor regulation and tissue regeneration. These findings highlight the potential of structural features alone to serve as effective regenerative cues, demonstrating a multi-level immunoregulatory strategy based entirely on the material’s intrinsic architecture. This approach offers a safer and more sustainable material-based solution for the treatment of inflammation-related diseases.

This study systematically demonstrates how changes in diameter scales serve as physical signals to remodel the macrophage immune factor secretion network, thereby regulating the immune microenvironment—from immune factor secretion and signaling pathway activation to *in vivo* tissue repair. Unlike traditional approaches relying on exogenous drug or biomolecule delivery, we propose an immune modulation strategy centered on the intrinsic scale and structural features of the material itself. This strategy transcends the conventional role of materials as passive carriers and establishes a new paradigm of structure-driven immune remodeling, offering novel theoretical insights and intervention strategies for regenerative therapies targeting chronic inflammation-related diseases.

## Conclusion

This study reveals that the micro- and nanoscale cues of fibers significantly influence immune factor secretion profiles. N-F with smaller diameter scales promotes the secretion of pro-inflammatory factors, while M-F with larger diameter scales enhances the expression of pro-regenerative factors. These findings highlight the potential of fiber diameter scale as a key factor in modulating the immune microenvironment, providing guidance for designing ‘immune-friendly’ fibers for tissue regeneration. The results offer valuable insights into the relationship between material structure and immune responses, paving the way for the development of advanced biomaterials with precise immunomodulatory functions for clinical applications in regenerative medicine.

## Supplementary Material

rbaf130_Supplementary_Data
