# Supplementary figures and images for: Influence of micro- and nanoscale cues on immune factors secretion: implications for immunomodulation
Source: Regen Biomater. 2025 Dec 18;13:rbaf130. doi: 10.1093/rb/rbaf130 (PMC12867575; doi:10.1093/rb/rbaf130)

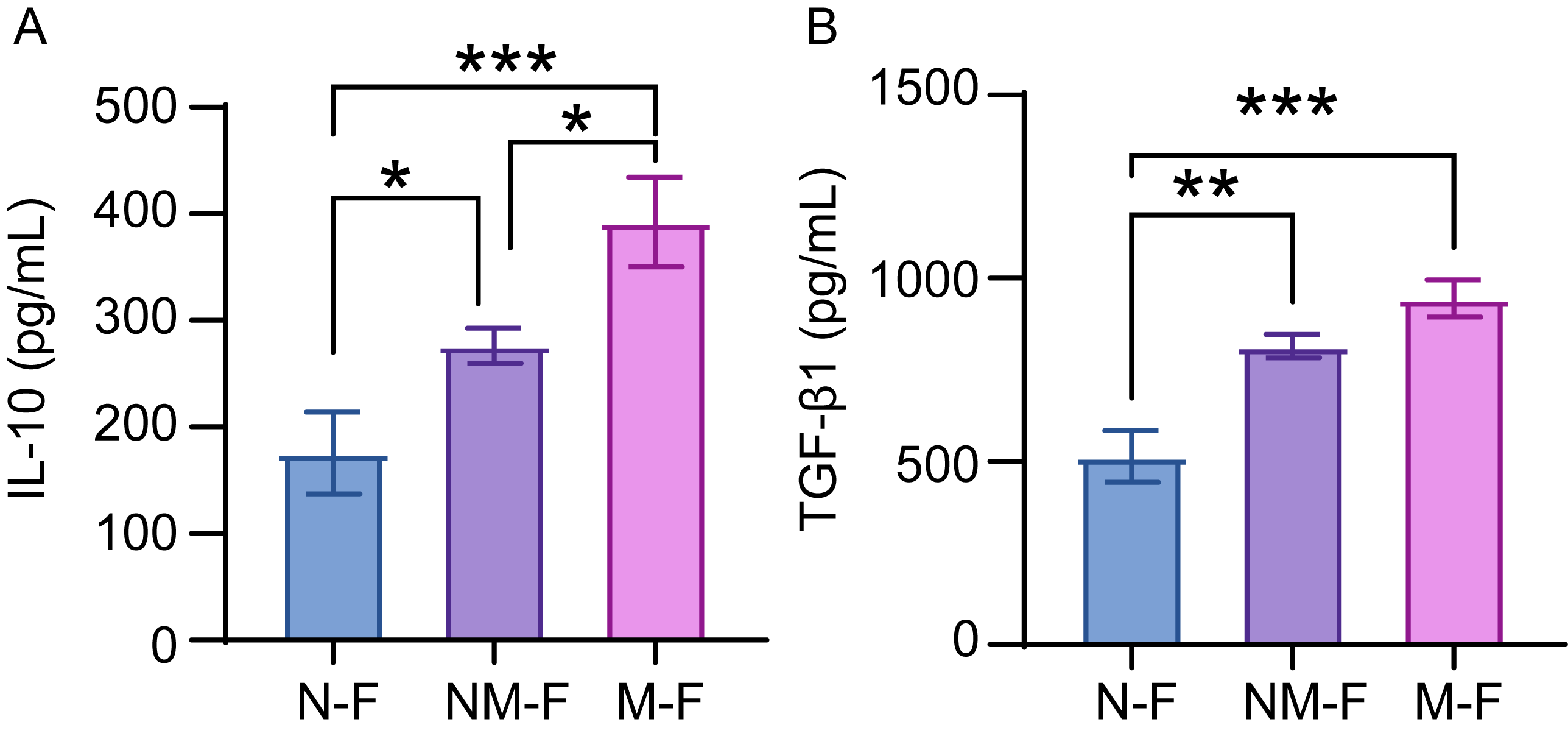


Figure S1. ELISA assessment of immune factor secretion. *P < 0.05; **P < 0.01; ***P < 0.001.

Supplement: rbaf130_Supplementary_Data [file rbaf130_supplementary_data.zip › RB-2025-416.R2-manuscript-Supplementary Figures-rev.docx]
